# Randomized controlled trial to evaluate the utility of suction and inner-stylet of EBUS-TBNA for mediastinal and hilar lymphadenopathy

**DOI:** 10.1186/s12890-018-0751-0

**Published:** 2018-12-07

**Authors:** Xiaoxiao Lin, Min Ye, Yuping Li, Jing Ren, Qiyan Lou, Yangyang Li, Xiaohui Jin, Ko-Pen Wang, Chengshui Chen

**Affiliations:** 1 Department of Pulmonary and Critical Care Medicine, The First Affiliated Hospital of Wenzhou Medical University, Ouhai District, Wenzhou, China; 2 Department of Pathology, The First Affiliated Hospital of Wenzhou Medical University, Ouhai District, Wenzhou, China; 3 Department of Endoscopy, The First Affiliated Hospital of Wenzhou Medical University, Ouhai District, Wenzhou, China; 4 Division of Pulmonary and Critical Care Medicine, Johns Hopkins University School of Medicine, Baltimore, USA

**Keywords:** Endobronchial ultrasound-guided transbronchial needle aspiration, Clinical trial, Lymphadenopathy, Malignant

## Abstract

**Background:**

The optimal procedure for maximizing the diagnostic yield and minimizing the procedural complexity of endobronchial ultrasound-guided transbronchial needle aspiration (EBUS-TBNA) is controversial. We conducted a prospective randomized controlled trial to determine the optimal procedure of EBUS-TBNA for mediastinal and hilar lymphadenopathy, with a particular focus on the roles of the inner-stylet and suction.

**Methods:**

Consecutive patients with enlarged mediastinal and hilar lymph nodes (LNs), detected by computed tomography (CT) or positron emission tomography-CT (PET-CT), who underwent EBUS-TBNA were included. Each LN was sampled with three needle passes using suction–stylet, suction–no stylet, and stylet–no suction procedures. The samples were smeared onto glass slides for cytological evaluation. A single, blinded cytopathologist evaluated each set of slides. The primary outcomes were cytological specimen adequacy rate and diagnostic yield of malignant LNs. The secondary outcomes were tissue-core acquisition rate, procedural time, and the amount of bleeding.

**Results:**

This study evaluated 97 patients with a total of 255 LNs. The final LN diagnosis was benign in 144, malignant in 104, and inadequate in 7 cases. There were no significant differences among the suction–stylet, suction–no stylet, and stylet–no suction groups in specimen adequacy rate (87.1, 88.2, 85.9%, respectively) or diagnostic yield of malignancy (32.2, 31.8, 31.0%, respectively). However, the use of suction was associated with an increase in tissue-core acquisition rate (*P* <  0.001). The no-stylet procedure decreased the average procedural time by 14 s (*P* <  0.001). There was no significant difference in the amount of bleeding among the procedures.

**Conclusions:**

The use of suction or non-use of an inner-stylet does not make a significant difference in cytological specimen adequacy or diagnostic yield when performing EBUS-TBNA. While omitting the stylet can simplify the procedure, applying suction can increase the tissue-core acquisition rate. These findings may assist endoscopic physicians in determining the optimal EBUS-TBNA procedure and warrant clinical verification in a future multicentre study.

**Trial registration:**

Trial registration: (ChiCTR-IOR-17010616). Retrospective registered date: 12th February, 2017.

## Background

Endobronchial ultrasound-guided transbronchial needle aspiration (EBUS-TBNA) is a technique that is highly effective for diagnosing enlarged mediastinal and hilar lymph nodes (LNs) detected on computed tomography (CT) or positron emission tomography-CT (PET-CT), in patients with benign or malignant conditions. Some clinical studies have demonstrated that EBUS-TBNA is a cost-effective [1] and safe diagnostic technique for acquiring specimens, with a diagnostic yield similar to or even higher than that of surgical mediastinoscopy [2–4]. However, the optimal procedure of EBUS-TBNA for maximizing the diagnostic yield and minimizing the procedural complexity is controversial. In the past decade, various modifications of the EBUS-TBNA procedure [5–9] have been described for optimizing diagnostic yield, procedural efficiency, and specimen adequacy. Nevertheless, there has been limited discussion on how to simplify the procedure without decreasing its diagnostic yield.

The conventional EBUS-TBNA technique requires that a metal stylet within the inner lumen of a fine needle be inserted and removed during every needle pass, which increases the procedural time and complicates the procedure. Therefore, we considered the possibility of omitting the inner-stylet during EBUS-TBNA. In addition, because there is considerable controversy regarding whether it is necessary to apply suction during EBUS-TBNA, we here conducted a prospective randomized controlled trial to determine the optimal EBUS-TBNA procedure for detection of mediastinal and hilar malignant lymphadenopathy, with a particular focus on the effect of using an inner-stylet and suction.

## Methods

### Trial subjects

Consecutive patients with enlarged mediastinal and hilar LNs who underwent EBUS-TBNA between October 2016 and May 2017 were enrolled. An LN with a short-axis diameter > 5 mm on a chest CT image was considered as an enlarged LN. Lymph-node stations were classified in accordance with the international lymph-node map by the International Association for the Study of Lung Cancer [10]. All patients provided written informed consent. The EBUS-TBNA procedure was performed by the same experienced endoscopic physician for all patients. It was performed if an enlarged LN was identified by using a convex-probe echoendoscope (EB-530, FUJIFILM, Tokyo, Japan). The trial protocol was approved by the Clinical Research Ethics Committee of our hospital (YJLCYJ2016–216), and the trial was registered at www.chictr.org.cn (ChiCTR-IOR-17010616).

### Trial procedure

TBNA was performed with a 22-gauge needle (NA-201SX-4022, Olympus, Tokyo, Japan) under EBUS and real-time color Doppler guidance with a convex-probe echoendoscope. After puncturing an LN, the fine needle was moved to and fro within the LN 10–20 times and then withdrawn. Each LN was sampled with three needle passes using suction–stylet, suction–no stylet, and stylet–no suction procedures (each process was performed once). To adjust for the effects of some potential confounding factors, including passes made using different procedures, the order of the procedure for each target site was randomized by a senior biostatistician from the School of Public Health, Wenzhou Medical University, using SAS 9.4 for Windows (Cary, North Carolina State, America).

During the conventional EBUS-TBNA procedure (the suction–stylet group), the inner lumen of the fine needle was first occluded with a metal stylet, which was removed after the needle entered the target LN. Once the stylet was withdrawn, a 20-mL syringe was applied to the needle for providing suction. For the no-stylet procedure, the stylet was omitted throughout the procedure, and the syringe was applied to the needle before the latter was inserted into the working channel of the echoendoscope. In the no-suction procedure, after the needle was inserted into the target LN, the stylet was withdrawn by 10 cm without using suction. During each pass, the physician assessed the amount of bleeding from the puncture site on the bronchial wall.

Each needle-pass specimen was extruded onto a separate glass slide using a 10-mL air-filled syringe, and a direct smear was made by an experienced EBUS nurse. Rapid on-site cytological evaluation was not performed. The residual contents of the needle from a single LN were flushed into the same container and consolidated by formalin to obtain a single cell block or tissue core for histological examination (in accordance with the handing and preparation procedures for histological specimens of our pathology department). After flushing the needle, the outside of the needle and the stylet were vigorously wiped with sterile gauze to reduce cross contamination between passes. A related on-site trial assistant recorded whether or not a visible tissue core was acquired with each needle pass and calculated the procedural time for each pass from the time of insertion of the fine needle through the working channel of the echoendoscope to the retrieval of the needle during each pass, excluding the time for reinsertion of the stylet into the fine needle. If insufficient specimen for a cell block or tissue core was obtained after the third pass, additional passes were permitted, with the choice of procedures and number of passes left to the discretion of the endoscopic physician.

The smears on glass slide were alcohol-fixed (95% ethanol) and stained with hematoxylin and eosin. A cytopathologist, who was blinded to the procedural order for EBUS-TBNA, characterized each individual needle pass for cytological specimen adequacy and made a specific diagnosis as follows: malignancy, benign (including normal lymphoid tissue and granulomatous inflammation), and inadequate. Specimens with > 40 lymphocytes per high power field [11] in the more cellular areas of the smeared slide was interpreted as adequate, as were those that exhibited malignant cells. Samples with insufficient diagnostic cellular materials or lymphocytes were deemed inadequate.

### Assessment of procedural outcomes

The final diagnosis of each LN was determined using all available cytological and histological findings from three EBUS-TBNA procedures. The primary outcomes of the trial were the cytological specimen adequacy rate and the diagnostic yield for malignant lymphadenopathy. A sample size of 225 LNs would provide sufficient power for a 10% non-inferiority margin upon performing non-inferiority analysis. The secondary outcomes were tissue-core acquisition rate, procedural time and amount of bleeding during each procedure. The amount of bleeding was categorized on the basis of the following scores: 0 (major hemorrhage, resulting in termination of subsequent procedure); 1 (light hemorrhage that could be stopped using cold saline or norepinephrine); 2 (no or little hemorrhage occurred, even without treatment).

### Statistical analysis

Statistical analysis was performed with the IBM SPSS Statistics Version 22.0 software (Chicago, IL, USA). The analysis evaluated only the first three passes for each LN. Descriptive statistics were used to summarize the characteristics of all patients and all LNs in the trial. Data on continuous variables were presented as mean ± standard deviation. McNemar tests were performed to determine the difference in specimen adequacy rate, diagnostic yield and tissue-core acquisition rate between suction–stylet and suction–no stylet or suction–stylet and stylet–no suction procedures. The paired t-test was employed to compare procedural time. In addition, the amount of bleeding was analyzed using Wilcoxon’s test. A subgroup analysis was performed to determine the association of different EBUS-TBNA procedures with specimen adequacy rate and diagnostic yield, for LNs >  10 mm or ≤ 10 mm in diameter. A two-sided *P* value of < 0.05 was considered to indicate statistical significance.

## Results

**Fig. 1:**
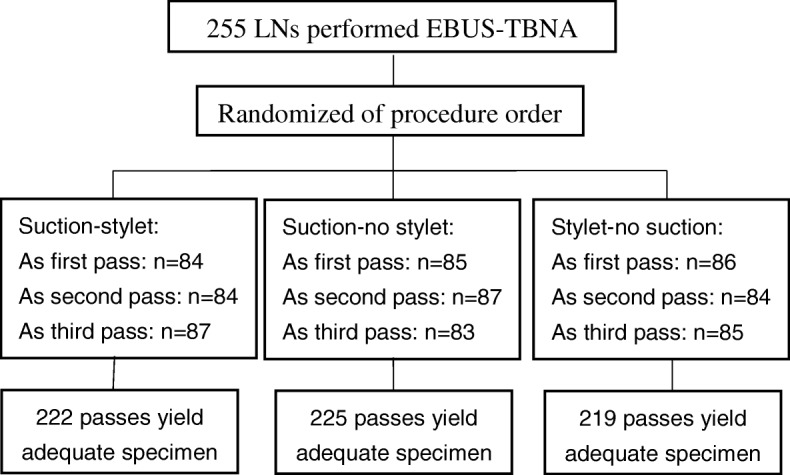
The number of each pass order and adequate specimen for different EBUS-TBNA procedures. EBUS-TBNA: Endobronchial ultrasound-guided transbronchial needle aspiration; LN: Lymph node

The trial evaluated 97 patients with a total of 255 mediastinal and hilar LNs. Randomization ensured that each third of the suction–stylet, suction–no stylet, and stylet–no suction passes were first, second, and third passed (Fig. 1). The baseline characteristics of the patients and LNs are summarized in Table 1. The mean age of patients was 61.2 years (range, 20–79 years); 71 (73.2%) of the patients were male. Of 57 inpatients for whom we could record clinical symptoms after the EBUS-TBNA procedure, 9 (15.8%) had transient fever; these 9 patients recovered within 24 h without treatment or with temporary antifebrile medication. There were no instances of procedure-related major hemorrhage in the present trial.

**Table 1:** Characteristics of patients and lymph nodes included in the final analysis

Characteristics	Data
Patients, No.	97
Age, years	61.2 ± 13.2
Gender, No.	
Male	71
Female	26
Origin of patient, No.	
Outpatient	40
Inpatient	57
Lymph nodes, No.	255
Mean nodule size, mm	14.7 ± 7.6
≤ 10 mm, No.	86
> 10 mm, No.	169
Location of lymph nodes, No.	
2 L	1
4 L	29
4R	72
7	77
10 L	6
10R	3
11 L	28
11R	26
12R	1
mediastinal mass	12

Two or three LNs were sampled for most patients. For the 255 LNs, the mean short-axis diameter on chest CT images was 14.7 mm, and the most common enlarged LN stations were 4R and 7. The final pathologic diagnoses for the 255 LNs were as follows: 104 malignancies, 144 benign diagnoses, and 7 inadequate samples.

The cytological specimen adequacy rates were 87.1, 88.2, and 85.9% in the suction–stylet, suction–no stylet, and stylet–no suction groups, respectively; the corresponding values for diagnostic yield of malignancy were 32.2, 31.8, and 31.0%, respectively, which showed no statistically significant difference among the three groups. The results of statistical analysis of specimen adequacy rate and diagnostic yield are detailed in Table 2. Subgroup analysis did not show a statistically significant association between the EBUS-TBNA procedures and specimen adequacy rate or diagnostic yield for LNs >  10 mm or LNs ≤ 10 mm (Table 3).

**Table 2:** Comparison of primary outcomes of EBUS-TBNA procedures

EBUS-TBNA procedure	The primary outcome	*P*^a^ value
	Specimen adequacy rate	
suction–stylet vs. suction–no stylet	87.1% vs. 88.2%	0.629
suction–stylet vs. stylet–no suction	87.1% vs. 85.6%	0.728
	Diagnostic yield	
suction–stylet vs. suction–no stylet	32.2% vs. 31.8%	> 0.999
suction–stylet vs. stylet–no suction	32.2% vs. 31.0%	0.711

^a^: Determined by McNemar test; *EBUS-TBNA* Endobronchial ultrasound-guided transbronchial needle aspiration

**Table 3:** Result of subgroup analysis among EBUS-TBNA procedures for LNs >  10 mm and ≤ 10 mm in diameter

Compared procedures	Subgroup	P^a^ value	
	LN size, mm	adequacy rate	diagnostic yield
suction–stylet vs. no-stylet	≤ 10	> 0.999	> 0.999
	> 10	0.754	> 0.999
suction–stylet vs. no-suction	≤ 10	> 0.999	0.289
	> 10	0.523	> 0.999

^a^Determined by McNemar test; *EBUS-TBNA* Endobronchial ultrasound-guided transbronchial needle aspirationm, *LN* Lymph node

Comparison of secondary outcomes among the three procedures (Table 4) revealed that the use of suction was associated with an increase in the tissue-core acquisition rate (suction–stylet vs. stylet–no suction group; 47.1% [120/255] vs. 32.5% [83/255]; *P* <  0.001). Non-use of the stylet did not decrease or increase the tissue-core acquisition rate (suction–stylet vs. suction–no stylet group: 47.1% vs. 49.4%; *P* = 0.576). In terms of procedural time, the no-stylet procedure was on average 14 s shorter than the suction–stylet procedure (87.1 s vs. 101.1 s; *P* <  0.001). However, in terms of amount of bleeding, the use or non-use of suction yielded similar scores; similar results were observed for procedures with and without the stylet.

**Table 4:** Statistical results of the three procedures in secondary outcomes

Secondary outcome	Group A^b^	Group B^c^	Group C^d^	P value^a^	
				A vs. B	A vs. C
Procedural time (second)	101.1 ± 31.3	87.1 ± 34.7	89.3 ± 33.6	< 0.001	< 0.001
Tissue-core acquisition (%)	47.1	49.4	32.5	0.576	< 0.001
The amount of bleeding (score)	1.97 ± 0.17	1.96 ± 0.20	1.97 ± 0.17	0.366	> 0.999

^a^Procedural time, tissue-core acquisition rate, and the amount of bleeding were analyzed by the paired t-test, McNemar test, and the Wilcoxon’s test, respectively

^b^A, suction–stylet procedure

^c^B, suction–no stylet procedure

^d^C, stylet–no suction procedure

## Discussion

This prospective randomized controlled trial demonstrated that the traditional procedure of applying suction during EBUS-TBNA did not make a statistically significant difference in cytological specimen adequacy or diagnostic yield of malignant lymphadenopathy, although it increased the rate of tissue-core acquisition for histological examination. Compared to procedures performed with a stylet, not using a stylet did not decrease the specimen adequacy or diagnostic yield. Upon comparing EBUS-TBNA procedures with or without suction and with or without stylet for LNs ≤ 10 mm and >  10 mm in short-axis diameter, we found no difference in the adequacy or diagnostic yield of cytological specimens. Although more data may be needed to confirm these specific conclusions, our findings may assist endoscopic physicians in determining the optimal EBUS-TBNA procedure.

Mediastinal and hilar lymphadenopathy may be caused by various inflammatory, infectious, or malignant factors, and it is important to ascertain the diagnosis or to determine the disease stage in case of malignancy before deciding on treatment. Mediastinoscopy has long been the reference standard of mediastinal and hilar LN sampling; however, it has several disadvantages, including its relatively high complexity and invasiveness [12]. Over the last decade, EBUS-TBNA has provided a more readily available and safer alternative than mediastinoscopy for acquiring specimens [2, 3]. It has emerged as the best first-diagnostic tool for collecting tissue for diagnosis and staging of lung cancer [13] and has also come to be approved for use in other lymphadenopathies, such as tuberculosis, sarcoidosis and lymphoma [14–16].

However, EBUS-TBNA has several limitations. Although its median sensitivity for detecting malignant lymphadenopathy (89%) [13] is better than that of imaging examinations alone, EBUS-TBNA leads to misdiagnosis of malignant LNs in an average of 11% of patients. Additionally, physicians need to obtain sufficient tissue specimens from EBUS-TBNA for molecular testing for diagnosis of malignancy or microbe cultivation for diagnosis of infectious diseases. Additionally, the EBUS-TBNA technique is time-consuming, especially when multiple LNs are identified and multiple needle passes are made. In our trial, we found that the procedure mainly required an additional 1–3 min for every needle pass and up to 10–15 min additionally per LN. Lastly, given the recommendations for combining EBUS-TBNA and endoscopic ultrasound-guided fine needle aspiration (EUS-FNA) for diagnosis of mediastinal and hilar lymphadenopathy [17], the operating physician should be skilled in both procedures. Consequently, it is important to simplify the EBUS-TBNA procedure and acquire adequate specimens without decreasing its diagnostic yield.

The use of an inner-stylet during EBUS-TBNA is somewhat controversial. It has commonly been used because it can theoretically prevent bronchial mucosa and cartilage filling the inner lumen and protect the fine needle by increasing its stiffness upon entry into the target LN. However, the inner-stylet has to be inserted and removed through the fine needle during every needle pass, which increases the procedural time and complicates the EBUS-TBNA procedure. Moreover, conventional TBNA is performed without an inner-stylet in the fine needle. Evaluation of the use of the inner-stylet in EBUS-TBNA has been limited to a single recent study. Scholten and colleagues [18] found no significant differences in diagnostic yield, specimen adequacy, or cytological quality between with-stylet and no-stylet procedures; these conclusions agreed with our findings. However, these previous authors failed to quantify the procedural time saved by omitting the stylet. In our trial, we found that non-use of the stylet statistically significantly decreased the procedural time; relative to the suction-stylet procedure, the no-stylet procedure was on an average 14 s shorter, excluding the time spent on inserting the inner-stylet into the fine needle. In addition, during the entire trial, there was no instance of needle breakage when the inner-stylet was not used. Moreover, from the patient’s perspective, omitting the inner-stylet might help reduce the cost of EBUS needles. Although the clinical value of the time saved with the no-stylet procedure merits further study, it is evident that omitting the stylet could simplify the EBUS-TBNA procedure, without reducing the cytological specimen adequacy or diagnostic yield.

Application of suction during FNA has been a standard practice for many decades in various medical specialties, including pathology and gastroenterology. However, there is considerable controversy about the need to apply suction during EBUS-TBNA. Some clinicians believe that suction might increase tissue trauma at the biopsy site and result in more bleeding into the specimen, thus decreasing the diagnostic yield of EBUS-TBNA. Others have argued that suction helps to acquire more specimen material. Wallace et al. [19] reported that, compared to FNA without suction, the traditional method of applying suction during EUS-FNA did not show any difference in diagnostic yield but provided worse specimen quality because of excessive blood in the specimen. Recently, Casal et al. [20] conducted a randomized trial for comparing the with-suction and no-suction procedures of EBUS-TBNA and found no difference in diagnostic yield, adequacy or quality of cytological specimens. However, they did not analyze the histological specimen adequacy of each procedure. A retrospective nonrandomized study showed that high suction pressures during EBUS-TBNA might be useful for safe collection of sufficient tissue specimens [21]. Our trial data support the conclusion that suction does not influence cytological specimen adequacy, diagnostic yield or the amount of bleeding.

Rapid advances in oncologic therapy have necessitated further ancillary studies, including immunohistochemical and molecular analyses for subtyping and genotyping of lung cancer, during the diagnostic workup of small tissue specimens. A guideline from the College of American Pathologists, International Association for the Study of Lung Cancer, and Association for Molecular Pathology, states that tissue samples should be prioritized for molecular analysis and that cytological samples are also suitable for studies, with cell blocks being preferred over smeared material [22]. In our trial, the suction procedure assisted in obtaining a greater volume of tissue specimens, which could be processed for cell block or tissue histology analyses. However, another guideline for the acquisition and preparation of EBUS-TBNA specimens for the diagnosis of lung cancer suggests that cell blocks and core tissue are both good materials for mutational analysis [23]. Numerous studies have reported on preparation of cell blocks for ancillary studies [24–26]. Therefore, further investigations may be needed to explore whether the amount of tissue-core obtained during EBUS-TBNA with or without suction would influence the diagnosis and subtyping of lung cancer.

The most frequent complication associated with EBUS-TBNA is hemorrhage; other rare complications of the procedure are infection, pneumothorax, and device breakage [27]. In 2014, a systematic review on adverse events in 16,181 patients who underwent endosonography for mediastinal and hilar LNs or central lung masses reported 23 (0.14%) serious adverse events (0.3 and 0.05% with EUS-FNA and EBUS-TBNA, respectively), with no mortality [28]. In the present trial, there were no instances of severe infectious disease, need for ICU admission, or death after EBUS-TBNA. The incidence of fever was 15.8% (9/57) among inpatients in our trial, and all febrile patients recovered in 24 h without treatment or with temporary antifebrile medication. In addition, there was no significant difference in the amount of bleeding with each pass between EBUS-TBNA procedures with and without suction or with and without stylet, and there was no instance of major hemorrhage. These results suggest that EBUS-TBNA is a safe method in general and that the probability of complications is similar among the different EBUS-TBNA procedures.

An advantage of the present prospective trial, which involved randomization of the procedure order and blinding of the cytopathologist, is its self-contrast design, which could control for the effects of size, location, density, and pathological type of different LNs, as well as other unknown factors. Furthermore, in our trial, an on-site assistant recorded the procedural time for each pass; this has not been evaluated in other previous studies.

A limitation of our trial, however, is its single-centre and single-operator design. A multicentre trial would be ideal to confirm the statistical significance of the results obtained with different EBUS-TBNA procedures. Additionally, we compared tissue-core acquisition rate among different procedures but, regrettably, failed to analyze the specimen quality for cell block or tissue histological examination, which might have decreased the chance of identifying differences among the procedures.

## Conclusions

In summary, the use of suction or non-use of the inner-stylet does not make a significant difference in cytological specimen adequacy or diagnostic yield when performing EBUS-TBNA. Omitting the stylet can simplify the procedure, and applying suction can help increase the tissue-core acquisition rate. These findings may assist endoscopic physicians in determining the optimal EBUS-TBNA procedure and warrant clinical verification in a future multicentre study.

## Abbreviations

CT: computed tomography
EBUS-TBNA: endobronchial ultrasound-guided transbronchial needle aspiration
EUS-FNA: endoscopic ultrasound-guided fine needle aspiration
LN: lymph node
PET-CT: positron emission tomography-computed tomography
